# Hospital and Institutional News

**Published:** 1917-08-04

**Authors:** 


### HOSPITAL AND INSTITUTIONAL NEWS.

### UNDESERVED AND ILL-FOUNDED CRITICISM.

Mr. Bishop Harman is reported in the *Daily Telegraph* as having stated, at the meeting of the British Medical Association, that "the governing bodies of the voluntary hospitals retained these men in blue" (wounded soldiers) "in the wards as an advertisement for their own hospitals." Dr. Robertson at once rose in protest, affirming that "the voluntary hospitals are doing their work from a grander and more patriotic point of view than advertising for more patients." Could more striking and forcible proof of the correctness of Dr. Robertson's view be desired than the following figures, showing the position of the work done at St. Thomas's Hospital in 1890 and in 1916? In the former year 4,881 in-patients were treated, whilst the number increased to 9,659 in 1916. The daily average number of beds occupied in 1890 was 358; in 1916 it was 789, including those in St. Thomas's Home. In 1890 there were 480 beds available, 90 additional beds being closed for want of funds; in 1916 there were 1,014 beds available for treatment of patients. The cases under treatment were:—Medical: male, 101; female, 70; surgical: male, 274; female, 70; children under six, 17; ophthalmic: male, 13; female, 12; obstetrics, 30; maternity (lying-in), 21; isolation, 70; soldiers' beds and soldiers' huts, 302; total, 1,014. There were 10 beds for tuberculosis, 10 for venereal cases, 10 for emergency (fever), and 40 for medical and surgical septic cases. The out-patients treated were: *x*-ray department, 6,959; medico-therapeutic, 16,225; maternity attendances, 5,586; tuberculosis attendances, 8,306; baby clinic attendances, 3,576; medical out-patients, 4,089; surgical, 230; massage patients, 1,303. In the face of these figures, which are one fair specimen of the work being done in war-time by the great voluntary hospitals throughout the country, Mr. Bishop Harman will see that he owes it to the profession and to himself either to prove his statement by the publication of irrefutable evidence, or to withdraw it promptly.

### PLASTIC SURGERY AT SIDCUP.

The Queen's Hospital at Frognal, Sidcup, which was open to inspection last week, has been established for the repair of facial and jaw injuries by means of plastic surgery. The hospital was started by Mr. C. H. Kenderdine, one of the originators of Queen Mary's Hospital at Roehampton. Fractured jaws often need considerable surgical and dental treatment before they are ready for plastic work. It is stated that the Queen's Hospital aims at the restoration of the features by means of surgical procedure, and that only in extreme cases will artificial features be used. A museum has been started at the hospital. Pastel drawings are preserved to show the condition of wounds before and after treatment. Casts are also shown. The success of the museum so far is largely due to the work of Professor Tonks and to the sculptor, Mr. Edwards. The special plastic work is undertaken by Captain H. W. Gillies and Captain J. L. Aymard and a staff of dental surgeons and mechanics. The commanding officer is Lieut.-Col. J. R. Colvin. A home farm and poultry farm form part of the institution, where the men, as they approach convalescence, can be pleasantly occupied. Since they suffer acutely from mental depression, this will be doubly valuable to them. No one who has not seen the drawings and models can realise how startling is the success gained in the reconstruction of features, even in cases apparently beyond hope. It is hardly too much to say that where the smallest foundation exists, and the word must be understood metaphorically, eventual success is possible. Hope need never be abandoned, and the most tragic cases of suffering have a chance which would not have been thought possible in pre-war days.

### THE DOOM OF THE QUACK.

In a deputation to the Local Government Board a few months ago the medical profession urged that quack treatment of venereal diseases should be prohibited, and it is to be prohibited. Lord Rhondda received that deputation, and almost his last act whilst President of the Department was to issue an Order under Section 1 (2) of the Venereal Disease Act, 1917, applying to the areas of certain counties, including London and county boroughs, section 1 (1) of the Act, prohibiting any person in such areas other than a qualified medical practitioner, for reward either direct or indirect, from treating any person for venereal disease or prescribing any remedy therefor, or giving any advice in connection with the treatment thereof, whether the advice is given to the person to be treated or to any other person. There is something emphatic about this. The Act also prohibits the issue of advertisements relating to the treatment of venereal disease, and from November 1, 1917, the issue of advertisements relating to the sale of preparations to be used as medicines or medicaments for the prevention, cure, or relief of any venereal disease is forbidden. This also is decisive. It is explained that the prohibition with regard to advertisements does not, however, apply to announcements by local or public authorities published with the sanction of the Local Government Board, or to publications sent only to duly qualified medical practitioners or wholesale or retail chemists for the purposes of their business.

### THE MILITARY OUT-PATIENT PROBLEM.

The Liverpool Council of Voluntary Aid informs us that, at a recent meeting of the Medical Charities' Committee of that Society, the treatment of service and ex-service men at the out-patient departments of the hospitals was considered. It was decided to refer the question, in order to secure uniformity of treatment, to the Lancashire County Advisory Committee (naval and military pensions), and to the British Hospitals Association. If the latter body will get quickly to work, which is not always easy, in view of the multifarious duties in different parts of the country in which the members of the Council are engaged, uncertainty should soon vanish, and a uniform method of procedure take its place. At the same meeting Major Macalister introduced a scheme for co-operation between the various Liverpool hospitals for the treatment of children's diseases. This co-operation is to be brought about by using the Invalid Children's Association as a connecting link between them. The two matters are worth placing side by side, since they prove the Liverpool Council's value in impartially encouraging co-operation in any suitable corner of the hospital field.

### THE UNFIT AND THE ARMY.

It is stated that Mr. Shortt's committee, not content with reforming the medical examination boards, is also determined to do all in its power to remove from the Army men who have been improperly passed as fit. To this end a return is to be asked for of all men who have been admitted to hospital within three months of being passed into the Army. Such a return should be the last argument in the long array which the committee has disclosed in favour of reforming the "system" of medical examination. It is now beyond cavil that the medical boards have been not only rushed and hurried, but have also been subject to various and varying military orders, with the result of saddling the country with an army of unfit men in hospital or various stages of breakdown. The expense and disaster so brought on the country and on individuals have been appalling. We hope that the new scheme which Mr. Shortt's committee has put forward will prevent any recurrence of it.

### THE AFFAIRS OF AUXILIARY HOSPITALS.

If the war had not relieved us of the faculty of wonder at mere immensity of numbers, the annual report on the accounts of Red Cross auxiliary hospitals at home would seem more startling than it does. The cost of maintaining 982 hospitals was £2,000,000. War Office capitation grants provided three-quarters of this sum, the remainder being derived from voluntary contributions. There are also, of course, a small number of these hospitals which are maintained entirely by voluntary contributions and receive no War Office grant. Where funds and generosity are capable of providing this independence elasticity is gained, since the institution is not bound by official regulations, some of which are as troublesome as official regulations are apt to be. In favoured circumstances it is possible for these privately managed hospitals to cost less than the official allowance, in spite of the fact that, other things being equal, the cost of an institution per bed varies inversely with the smallness of its size. In the big hospitals the bills for surgery and the dispensary are naturally higher, since the more serious cases are handed over to them.

### DOCTORS AND TAXI-CABS.

When taxi-cab drivers are churlish, as they often are, or drive past one with serene indifference to any claim upon their attention, or say that they can only go in a south-westerly direction, or absolutely refuse to drive to Hampstead, a humane person is at a loss to know whether they are arrogant, or have a legitimate reason for their condition or refusal. To doctors, who expect to be driven anywhere, these doubts do not occur, and as a result of a summons a taxi-cab driver was fined 40s. in the Marlborough Street Police Court for "a sulky, deliberate, churlish refusal to do his duty." It was late at night when three doctors in Dover Street asked the driver to take them to St. Bartholomew's Hospital. He told them to leave the cab, and when they replied by calling upon a policeman he declared that he had no petrol. It was stated in court that he had really five and a-half inches in his tank. The magistrate told him that if he was summoned again he would no doubt be deprived of his licence. The man in the street will probably take the hint and describe himself as a doctor the next time a cabman refuses to carry him.

### THE PRINCE OF WALES' HOSPITAL FOR LIMBLESS SAILORS AND SOLDIERS, CARDIFF.

The half-yearly meeting of the Council of the Prince of Wales' Hospital, Cardiff, was held at No. 10 Downing Street, London, on Tuesday, July 24, under the presidency of Mrs. Lloyd George. There were also present the Lord Mayor of Cardiff (Councillor J. Stanfield, J.P.), Lord Aberdare, Lieut.-Col. J. Lynn Thomas and Mrs. Lynn Thomas, Surgeon-General M. W. Russell, C.B., Mr. and Mrs. Percy Miles, and the secretary, Mr. Gilbert D. Shepherd. The executive committee reported that the alterations and equipment were not yet fully completed, and the date of the formal opening was left, therefore, in the hands of Mrs. Lloyd George. A portion of the garden measuring about 100 ft. by 40 ft. is being laid out on a new plan, in order to assist the men fitted with artificial legs in the art of walking up and down various gradients and on different sloping paths. Most of the patients in this hospital are ordinarily resident in different parts of Wales, so that it is highly important that they should be taught to walk upon other ground than that of an ordinary level parade ground. Three large mounds are being built up in the garden, two of them 6 ft. high and the other 5 ft. Winding paths are being made with varying gradients from one in twelve to one in three. These paths will incline from one side to the other, some being from right to left, and the others from left to right. It is evident that this special training ground, with its miniature hills and dales, will enable the men to return to their homes with greater confidence in their artificial limbs to tread their native mountains and valleys.

### A MEDICAL REVOLT.

At the last meeting of the Health Committee of the Bournemouth Corporation, a letter was received from Mr. Robson Burrows, the chairman of the Royal Victoria and West Hants Hospital, to the effect that since receiving the draft agreement between the Corporation and the Hospital for Maternity Cases, a difficulty had arisen in view of the fact that the honorary medical and surgical staff of the hospital objected on principle to perform work, for which it was contended the Council should pay, which is authorised by the Local Government Board, and which is outside the scope of their ordinary duties. Having received a report of the medical officer of health on the question, the committee recommended that the Town Clerk should confer with Mr. Burrows and the medical officer of health on the points raised.

### LIGHTENING THE MEDICAL DUTIES AT BERMONDSEY.

The Bermondsey Board of Guardians have adopted a scheme placed before them by the medical superintendent at the infirmary (Dr. E. S. Bell), with a view to assisting the depleted medical staff. They have decided to call in the services of local medical practitioners in cases of emergency amongst the outdoor poor. On four afternoons a week there will only be one medical officer on duty at the infirmary, available for these outside cases, and consequently, if the possibility should arise that he was engaged on a case in the infirmary that he could not leave, it would be to the best interests of the outdoor sick poor if they were attended by a local doctor up to the time of their admission to the infirmary; the doctor will be paid a fee for his attendance, and also for issuing an admission certificate. Dr. Bell made the suggestion owing to his anxiety not to have any case neglected either by himself or the medical staff. There is little doubt that the great majority of the local practitioners will be only too pleased to assist the infirmary staff in this their hour of need and trial.

### AUSTRALIAN DOCTORS AND COMPULSION.

The Australian Branches of the British Medical Association have recently had a referendum on the question of the desirability of legislation for the compulsory enlistment of the medical profession in Australia for service at home or abroad. 1,361 doctors voted, and the voting resulted in a majority of 661 in favour of compulsion. The vote is extremely interesting and demonstrates, if any demonstration were needed, the patriotism of the medical profession in Australia, and it cannot fail to have much influence on the question of proposals for the mobilisation of the entire medical profession in Australia.

### THE SOLDIER'S MOTHER.

One of the problems of the war, which the Poor Law has been asked to solve, is the financial position of a soldier's mother when her son marries and thereby dispossesses her of the separation allowance and supplementary payments which are transferred to his wife. That the wife is entitled to them is beyond dispute. But that the mother has a claim in ethics, if not in law, is also evident. Could not, it is suggested, the War Associations who give this supplementary allowance transfer it in these cases? The plan is a bad one, because if the mother could not live on the separation allowance it is probable that the wife cannot do so either. But the worst plan of all is to send the soldier's mother to the workhouse or allow her to receive Poor-Law relief. If she was dependent on the soldier before his marriage, she is so still. The soldier by his marriage has added to the number of his dependants. When these are children the law recognises his claim to an increased pension. When the mother becomes the additional dependant, it does not. The simplest plan would be for a pension to be given to her as well. If, as is to be feared, the Pensions Ministry has no power to do this, there is an organisation at hand to relieve it of the privilege. That organisation is the Prince of Wales' National Relief Fund. This obstructive and mismanaged body spends most of its time in reducing itself to impotence with red-tape. If its managers were the only victims, no one would regret their suicide. But when the soldier's mother is the type of its many victims, this is not to be borne. Why should not H.R.H. apply the necessary broom? It would make him popular with the people for all time.

### MANCHESTER LOCK HOSPITAL'S NEW SCHEME.

With the beginning of August the Manchester and Salford Lock Hospital enters on a new sphere of usefulness by having been approved by the Local Government Board as an institution for the diagnosis and treatment of venereal diseases. It will be open to inspection by the Board's officials at any time, and its committee will have to notify any proposed change in the medical staff. On these conditions the arrangements for carrying out the scheme will be made at the cost of the local authority, to which the Board will repay 75 per cent. of the outlay incurred. At present the hospital has added four more beds, an operation theatre, and a consulting-room, thereby improving on the tradition under which men were not admitted as in-patients, and the majority of patients of either sex were relegated to the out-patient department. The medical officers already approved are Dr. A. J. Selwards and Dr. Alexander Wilson, with Dr. W. J. S. Reid as their temporary assistant.

### HOSPITALS ON SHIPS.

The provision of hospital or isolation accommodation on ships is urged in the annual report of the medical officer of the Port of Liverpool. The occurrence of cases of infectious illness on shipboard, such as smallpox and enteric fever, renders it a most urgent matter, and is an important point to be borne in mind when planning new ships. The medical officer also declares it to be desirable to make some provision for the preservation of vaccine lymph on board vessels. It is well known to ships' surgeons and others that vaccine lymph rapidly deteriorates on shipboard, especially when the vessels pass through tropical climates, with the result that when smallpox breaks out, as it not infrequently does, the lymph is absolutely useless to protect the remainder of the crew or passengers. It is well known that vaccine lymph will keep indefinitely when preserved at temperatures below the freezing point, and such temperatures could easily be obtained on board most of the larger vessels, where some form of cold storage is installed for various purposes. Vessels trading in the tropics, too, should be fitted with appliances to guard against the entrance of mosquitoes into the living quarters. These insects in certain specified areas can transmit malaria and yellow fever. In addition to a hospital, a ship should have a small disinfecting apparatus. This is the case in some large ships, but there can be no doubt that a small disinfector of a suitable type would prove generally useful.

### RETFORD HOSPITAL TO BE REBUILT.

Retford is to have a new hospital. Colliery developments which are contemplated in the neighbourhood and other local requirements have impressed a sub-committee appointed to investigate the question with the need of a better building. The present premises served excellently in the past, but they have become out of date, and they do not admit of advantageous enlargement. Nor is their situation regarded as desirable. Therefore it has been decided to erect a new institution in a six-acre field on the North Road, the owner, Major Denman, having generously offered to give the site to the hospital authorities. A subscription list has been opened, and among the larger amounts already received are £250 from Mr. R. Eddison, £200 from the Mayor and Mayoress, and £100 each from Sir Charles Ellis, K.C.B., Mr. C. W. Kayser, and Mr. and Mrs. V. S. Woods. The sale of the existing premises and site is expected to realise a substantial sum towards the cost of the new hospital.

### THE PILKINGTON SPECIAL HOSPITAL.

This institution, which was founded and is supported by Messrs. Pilkington, of St. Helens Glass-works, claims to be the first to have instituted the combined treatment of the disabled. It employs the four methods of massage, whirlpool baths, electric treatment, and the French system of mechanotherapy, the success of which is stated to have been sufficiently proved for ten similar institutions to have been established under the War Office. Its beds number 135, and the staff, under Dr. James Kerr, includes a matron, fourteen masseuses, and fourteen nurses. It also possesses an out-patient department where between twenty and thirty discharged soldiers are under treatment.

### THE CYRIL CLAYDEN BED.

An admirable method of showing their esteem and their appreciation of the services of a trusted employee has been adopted by the great shipowning firm of Messrs. Furness, Withy and Co. Mr. Cyril Clayden, of Saffron Walden, died suddenly while acting as a superintendent and marine engineer for them, and the firm, in order to perpetuate his memory, has contributed £1,500 for the endowment of a "Cyril Clayden" bed in the Saffron Walden Hospital. This is an idea which will doubtless commend itself to other big undertakings when they lose the services through death of men whose memory they desire to keep green.

### MORTALITY IN WAR HOSPITALS.

The London County Council has prepared short reports on the two war hospitals it has equipped at Horton and Manor. The feature of interest is the low death-rate. As it is not possible to gather such information from other war hospitals it is interesting to mention the figures at these two County Council hospitals. Horton, opened in May 1915, has a record of 242 officers and 21,887 men admitted, and the deaths have been only two officers and 166 men. At the Manor Institution, where 3,334 patients have been admitted since the opening last October, the deaths have been six. At Horton the maximum accommodation is 2,532 beds, whilst at Manor there is accommodation for 1,200. Originally Manor was classified as a secondary hospital, but since May it has been declared to be a primary or general hospital, and, at the request of the War Office, one ward is being set aside for about forty officer patients.

### SOLDIERS AND FANCY-WORK.

It is well known that the "handy man" of the sea is something of an adept with his needle, not only in connection with the duties of his calling in the matter of the stitching of sail-cloth, but also in the making and repairing of his clothes; but it is probably not generally known to what a great extent sailors and soldiers in hospitals are users of the needle, more especially in the production of work of a fancy nature. This work appears, from all accounts, to be a very real and valuable form of recreation. Some excellent examples of drawn-thread work are often to be seen in many institutions, which one learns are the work of former patients; the working of what our grandparents were pleased to call "samplers" is another favourite piece of work, and usually takes the form of the soldiers' regimental crest or else something connected with the hospital which shelters him, while "fancy work," woollen slippers and waistcoats, and baskets of chip and rush are all to be seen within the walls of the many military, naval, and Red Cross hospitals which are dotted about the country to-day.

### THE MANAGEMENT OF SANATORIA.

Readers of the articles on this subject published in The Hospital will be familiar with our exposure of the current mismanagement of public sanatoria as evinced by the insecurity of tenure experienced by sanatorium medical residents. Yet another instance of the kind is lately reported where an experienced medical superintendent, a medical consultant of some note, and, it is said, a very prominent member of the committee of management, are resigning more or less voluntarily, largely as a result of disagreement with a subordinate lay official, and very much to the regret of the patients. What adds significance to the affair is that the previous medical superintendent was practically dismissed. This sort of thing is the very ruin of British tuberculosis work, and one of the chief reasons, carefully hidden though it be, why this country is so backward in phthisiology. The remedy is so important that we venture to repeat it. It is improvement of the status of the sanatorium medical superintendent so that his position equals that of the superintendent of an asylum for the insane. In all probability this can be done only by the system of centralised and specialised control of the anti-tuberculosis campaign, as was recommended by Sir William Osler.

### TUBERCULOUS MILK IN LONDON.

The prevalence of tuberculosis in milch cows is shown by the results of the examination of milk samples obtained by the London County Council's inspectors during the year 1916. In all 1,160 specimens were examined bacteriologically by the Lister Institute, and of these 101 showed the presence of tubercle bacilli—that is, 8.7 per cent., a rather higher percentage than in previous years. In connection with the samples the Veterinary Inspector visited 106 farms and examined 3,570 cows; of these, 70 cows showed signs of tuberculosis or were in some other way out of health. In the case of each cow suffering from tuberculosis the farmer agreed to have it slaughtered. There were also inspections of the cows kept in the 154 London cowsheds. No cases of tuberculosis of the udder were detected, but in 76 cows diseased conditions of the udder were found. Five cows showed signs of pulmonary or general tuberculosis, and these were slaughtered or otherwise disposed of by the owners.

### FERRARY'S HOSPITAL STAMPS.

The recent death of the famous stamp-collector, Philippe la Rénotière von Ferrary, in Lausanne, recalls the fact that his collections, on which about a quarter of a million had been spent, includes among its rarities, we believe, no hospital stamp of special note. On the Continent these stamps have long been popular, and now our Colonies have adopted the principle of the practice by issuing certain of their stamps with a surcharge of 2 cents for the benefit of the Red Cross. The recent ones issued by the Straits Settlements are marked in black "Red Cross 2c.," and some particulars concerning them were given by Mr. F. J. Melvill in a recent issue of the *Daily Telegraph*. Hospital men can still remember the stamps issued for the benefit of hospitals twenty years ago, and the fact that they did not endure was as much due to official opposition as to the Englishman's hatred of covering his correspondence with coloured devices of this kind.

### BRIGHTON AND CHILD WELFARE.

"Strike the iron while it is hot" is an old proverb with much truth in it. Some members of the Brighton Corporation are firm believers in it; they have just put it to the test and gained a signal success. Had it not been for the recent National Baby Week a proposal to appoint an assistant medical officer for the work of the ante-natal clinic at a salary of £350 would have been pooh-poohed all round, and the chances of securing the necessary majority would have been very remote. But within three weeks of Baby Week the appointment of such an officer has been confirmed. Like everything else in these grim war days a good deal of the work appertaining to child welfare at Brighton has been allowed to remain dormant, but the urgent need of the Corporation grappling with it was aroused by the interest taken in Baby Week, coupled with the demand which is being made upon the manhood of the country. Notwithstanding the paramount importance of the subject—a subject not considered half enough by many local authorities—the vote of the money met with some opposition. Some people seem so obsessed with the idea of economy —not only in war time—that they are unable to breathe the atmosphere of advancement even when its absolute necessity is brought right home to them as is this subject of child welfare. Fortunately, the opposers were in the minority. They advanced all manner of obstacles simply with a view of hampering the scheme, or maybe to catch a few supporters. They were anxious to know what would follow: did it mean that it would involve new buildings? The majority replied by agreeing to send Dr. Duncan Forbes, medical officer of health, and the chairman of the Health Committee to Bradford to see what is being done there. Brighton is to be congratulated on the step it has taken. It will be found that the conservation of infant life, whatever it may cost, will be a gilt-edged investment in the long run.

### HAVE THE BLIND A SIXTH SENSE?

The board of management of Henshaw's Blind Asylum, Manchester, secured the attendance of an interesting figure at their annual meeting last week, in Mr. A. W. G. Ranger, chairman of the National Institute for the Blind. Mr. Ranger, who has been blind from boyhood, is a Master of Arts and is stated to be the only blind man who has won the coveted degree of D.C.L. He is a solicitor and has practised for over thirty years. In the course of an address, Dr. Ranger said it was erroneous to believe that the blind possessed a sixth sense. This supposed sixth sense would be found, if examined, to be due to a somewhat exceptional training in the use of the four senses, making them co-operate immediately and carry unerringly a correct impression to the brain. The asylum's annual report showed that the expenditure last year exceeded the income by £1,190, the public subscribing only about £200. This is indeed a paltry sum in view of the splendid work done by the institution. The number of persons receiving benefit from the charity is 455. Lord Derby was elected president.

### ADMISSION TO ISOLATION HOSPITALS.

There has been a good deal of doubt as to the rules for the admission of cases into isolation hospitals, and the Urban District Councils' Association has been considering the question. A letter was received from the Local Government Board on the matter, and the Alton Urban District Council wrote to the Association asking for help in the interpretation of the powers of the hospital authorities. The Parliamentary Committee of the Association carefully considered the question, and came to the conclusion that all cases sent to an isolation hospital on the certificate of a medical practitioner should as a general rule be admitted, if the hospital is not full, and that refusal would not be justified unless the medical officer of health was of opinion that there were reasons which rendered it desirable that home isolation should be provided for the particular case.

### HEALTHY LONDON.

DR. W. H. HAMER, the medical officer of health for the London County Council, has issued his report for the year 1916, and in it he states that, on the whole, the health of London during the year compares very favourably with that of the preceding three months. 1916 was very wet, and we agree with the opinion he expresses that this fact must have had great influence on the health of the people of London. The influence is chiefly seen in the reduction in the extent of epidemic throat diseases, and in the occurrence of enteric fever and epidemic diarrhœa. There is another point in which the year has been unlike those preceding it; the great mass of the people has been well nourished, for wages have been high, though of course the price of food has been high also. The restrictions on the sale of alcohol must also have had no little effect, and probably some influence in the reduction of infectious diseases must be attributed to the restrictions on communications and traffic, especially with places abroad. When the war comes to an end, it is probable that the spread of infectious diseases will be much favoured in this connection. It is to be noted that the Sanitary Service is at present much depleted, and it is of the utmost importance that when the war is drawing to a close great attention should be paid to the reinstatement of the full Sanitary Service. There is one element in the estimation of the percentages of sickness in London that is doubtful, and that is its population. The Registrar-General estimated the population of the County of London in the middle of 1916 at 4,237,387, but Dr. Hamer believes that this is an underestimate. The number of cases of enteric fever during the year was 461, while in 1915 there were 607; but the figures are not strictly comparable, as military cases were not included in the same way in the two years. Yet Dr. Hamer points out that in recent years there has been a great and a progressive decline in the number of enteric cases, and he attributes this almost entirely to the diminution in the consumption of shell-fish and fish from polluted sources, and with the removal of "layings" of shell-fish from the neighbourhood of sewage outfalls. During the year there were 822 deaths from measles, as compared with 2,286 in 1915; and in this connection it is of interest to note that the compulsory notification of measles commenced on January 1, 1916. There has also been a very great decrease in the number of cases of scarlet fever, and Dr. Hamer is inclined to think that the dust from the explosion of shells may have been in part responsible for this reduction.

### THE "SEARCHLIGHT" AND "SOUTHERN CROSS."

THE July number of the *Southern Cross* may well welcome the appointment of Lieut.-Colonel J. E. H. Sawyer to the post of Administrator of the 1st Southern General Hospital, Birmingham. He has been a constant contributor to the *Southern Cross*, and as the hospital's historian has done something to make its pages of permanent value and interest. He was, previous to his promotion, registrar, and has been associated with the hospital ever since it was established nine years ago. "M.R." makes excellent fun of certain signatures which appear on the patient's "boards," as can be guessed when, as a character-reader and calligraphic expert, he remarks that total illegibility indicates a unique degree of modesty and self-effacement. We have not seen the comic side of medical handwriting unfolded before in these gazettes, and remark on it as showing what capital articles can be written on the every-day trivialities of hospital life. But then it takes a wit to see them. In the July *Searchlight* there is a rather humorous full-page drawing, in which the imaginary small boy of the future is seen asking his grandfather what he was doing during the war. The grandfather, with the help of his crutches, first turns his chair into a mimic stretcher, then goes down on his knees and pretends to scrub the floor; then he puts one crutch over his shoulder and seizes a pail. The child, appalled at these austerities, very justly asks, "What medal did they give you for all this?" "They gave me the Red Cross," the old man replies. This is equally good as sentiment or irony.

### THIS WEEK'S DRUG MARKET.

THE difficulty of obtaining adequate supplies of some of the more commonly used vegetable drugs is becoming more pronounced, and as a consequence extreme prices are asked for what is available. Cape aloes is very scarce and dearer, and the same may be said of buchu leaves, cascarilla bark, gamboge, sarsaparilla, opium, and various other drugs. In synthetic drugs there are very few price changes, but values on the whole are firmly maintained. Salicylates are still in short supply, but the advance in price appears to have ceased for the time being; barbitone is slightly more plentiful, but supplies are far from sufficient to meet the demand. The position of bromides is unchanged. The extreme prices still quoted for cod-liver oil restrict business within very narrow limits. Cream of tartar is again dearer. Supplies of sugar of milk are very small, and the price continues to advance. A small business is being done in quinine at slightly higher rates. The price of potassium permanganate is not quite so firmly maintained, as buyers are unwilling to do business at the extreme rates quoted by holders. Honey is again cheaper, and supplies are plentiful.

### TO OUR READERS.

**CONTRIBUTIONS are specially invited from any of our readers to these columns. They should deal with topical subjects and news. They must be authenticated for the information of the Editor only. The minimum payment if published is 5s.** There is no hard-and-fast rule as to space, but notes of about twenty lines in length are preferred.

